# The Role of Mycophenolate Mofetil for the Induction of Remission in ANCA-Associated Vasculitis: A Meta-Analysis

**DOI:** 10.3389/fmed.2021.609924

**Published:** 2021-03-01

**Authors:** Anji Xiong, Chen Xiong, Guancui Yang, Yu Shuai, Deng Liu, Linqian He, Zepeng Guo, Liangwen Zhang, Yi Liu, Yuan Yang, Beibei Cui, Shiquan Shuai

**Affiliations:** ^1^Department of Rheumatology and Immunology, Nanchong Central Hospital, The Second Clinical Medical College, North Sichuan Medical College, Nanchong, China; ^2^Department of Rheumatology and Immunology, West China Hospital, Sichuan University, Chengdu, China; ^3^Department of Rheumatology and Immunology, The First Affiliated Hospital of Chengdu Medical College, Chengdu, China

**Keywords:** mycophenolate mofetil, ANCA-associated vasculitis, cyclophosphamide, efficacy, meta-analysis

## Abstract

**Objectives:** The successful introduction of mycophenolate mofetil (MMF) as a treatment for renal allograft reduced the incidence of acute rejection. The inspiring effects obtained by the MMF have led to an evaluation of its therapeutic potency on ANCA-associated vasculitis (AAV). However, there is little evidence of the MMF's efficacy on the AAV. The meta-analysis is carried out to evaluate the efficacy of MMF as a remission induction therapy in AAV.

**Methods:** Up to June 30th, 2020, PubMed, Cochrane Library, and Embase have been searched comprehensively. According to heterogeneity, the pooled remission rates are synthesized by either fixed-effect or random-effect models.

**Results:** The eight included studies comprising 230 patients who were treated with MMF as induction therapy are included in our analysis. The pooled overall remission rate is 74% (95% CI: 0.68–0.80). The remission rate, the infection rate and the rate of leukopenia of four randomized controlled trials aimed at comparing the effects of MMF with cyclophosphamide (CYC) during induction therapy for AAV have no statistical significance (*P* > 0.05).

**Conclusion:** MMF may be an alternative to CYC for remission induction therapy in AAV with MPO-ANCA, mild to moderate renal involvement and non-life-threatening state. Whether to observe the effect of MMF in AAV or to compare the difference between MMF and CYC in the future studies, risk stratification and subgrouping of AAV patients should be first carried out to correctly identify the AAV subgroup suitable for MMF.

## Introduction

Anti-neutrophil cytoplasmic antibody (ANCA)-associated vasculitis (AAV) was a rare group of autoimmune diseases of unexplained reason characterized by inflammatory cell infiltration leading to vascular necrosis (1). A study conducted by watts et al. (2) reported the incidence and prevalence of AAV that both of them had generally increased over the last 20 years. Treatments for induction of remission and maintenance of remission were required because of its remission and relapse in AAV (3).

Cyclophosphamide (CYC) has been widely used in induction therapy for AAV for decades with a remission rate of 70–90% (4, 5). However, CYC leads to many serious acute side effects, such as haemorrhagic cystitis, tumors of the urinary bladder, infertility, and bone marrow depression (6). Hence, exploration of new agents with similar efficacy and less toxicity is needed.

The successful introduction of MMF as a treatment for renal allograft reduced the incidence of acute rejection (7). MMF has been used to treat immune-mediated kidney injury because it retards lymphocyte proliferation (8, 9). And unlike CYC, MMF is irrelevant to urothelial malignancy or infertility (10). AAV is a heterogeneous group of inflammatory diseases that affects the small vasculatures in multiple organs, but primarily the kidneys (11). MMF has been used as a remission induction therapy in AAV patients with CYC failure or intolerance (12–15).

A Brazilian guideline (16) recommended CYC, rituximab (RTX), methotrexate (MTX) and MMF to treat AAV. MMF had no reproductive toxicity of CYC, was cheaper than RTX and less hepatotoxic than MTX. However, only several small open-label studies (11, 17–19) compared the use of MMF to CYC in AAV induction therapy. The results of Hu et al. (17) showed that MMF led to a lower Birmingham Vasculitis Activity Score (BVAS) value compared with CYC for remission induction therapy in AAV and other three studies (11, 18, 19) found no difference between MMF and CYC in microscopic polyangiitis. There is no enough evidence to recommend the use of MMF in the induction therapy of AAV, thus, we conduct a meta-analysis to evaluate the role of MMF for the induction of remission in patients with AAV. Besides, two recently published meta-analyses (20, 21) have studied the randomized controlled trials (RCTs) comparing MMF with CYC. They reached conclusions that there was no difference in the therapeutic effects of MMF and CYC for AAV patients (20), and MMF was equivalent to CYC (21). Our article will further discuss the results of related studies.

## Methods

### Materials and Methods

We conduct a meta-analysis following the methods specified in the Cochrane Handbook for Systematic Reviews of Intervention (22). Eligible trials are identified through electronic searches (conducted by two independent reviewers, Guancui Yang and Deng Liu). PubMed, Cochrane Library and Embase are comprehensively searched up to 30 June, 2020 for the pertinent studies. Medical Subject Headings (MeSH) terms or free text are used as follows: “Mycophenolate Mofetil” or “Mycophenolic Acid Morpholinoethyl Ester” or “Cellcept” or “Mycophenolate Sodium” or “Myfortic” or “Mycophenolate Mofetil Hydrochloride” or “RS 61443” and (“Antineutrophil Cytoplasmic Antibody Associated Vasculitis” or “ANCA-Associated Vasculitis” or “Pauci-Immune Vasculitis” or “Wegener's granulomatosis” or “Microscopic polyangiitis” or “Churg-Strauss syndrome.” In addition, the clinical trials registry (ClinicalTrials.gov) is searched to obtain information on the registered clinical trials. Moreover, we scan reference lists of qualifying studies to identify other relevant studies.

### Selection Criteria

We first perform an initial screening of titles or abstracts. A second screening is based on full-text review. Studies written in English or Chinese are included in this meta-analysis if they meet the following conditions: (a) patients with AAV disease diagnosed based on positive or histologically proven ANCA disease; (b) studies demonstrating information of MMF for the induction of remission of AAV; (c) with or without a control group prospective or retrospective studies; (d) outcomes assessed by the overall response (OR). Exclusion criteria are as follows: case report for <5 patients, review, editorial, or meeting summary. All reported adverse events are included for safety assessment. Remission is defined as the absence of manifestations attributable to active disease [(BVAS) = 0)] and C-reactive protein <10 mg/dl. Relapse is defined as the reoccurrence or new appearance of organ involvement attributable to active vasculitis and requiring increase in or reintroduction of immunosuppression. Resistance is defined as a progressive decline in kidney function with persistence of active urine sediment, or persistence or new appearance of any extrarenal manifestations of active vasculitis despite immunosuppressive therapy.

### Date Extraction

Relevant data are extracted by two reviewers (Yu Shuai and Linqian He) according to the predefined scheme. And they carefully extract the following characters: country, study design, disease category, follow-up months, numbers of patients with remission in the treatment and so on. Two authors (Yu Shuai and Linqian He) independently conduct the data extraction. Any disagreements are resolved by discussion.

### Methodological Quality Assessment

Corresponding binomial parameters are used to determine the quality score of each study based on previous reports (23, 24). Each parameter receive a numerical score of 0 or 1, with an overall quality score ranging from 0 to 10. Studies with a ≥5 quality score are rated as high, while those <5 are rated as poor. This quality assessment is detailed as shown in Supplementary Table 2.

### Statistical Analysis

The estimated pooled remission rate is calculated through Log transformation (25). The *I*^2^ test is used to evaluate the statistic heterogeneity (26). Heterogeneity values of 25, 50, and 75% is designated as low, moderate and high. If heterogeneity existes, random effect model is used to assess the pooled rate and 95% confidence interval (CI), and if not, they would be assessed by fixed effect model (27, 28). The source of heterogeneity is detected by meta regression. Sensitivity analysis is performed to evaluate whether any single study influence the overall results in order to confirm the stability and liability of the meta-analysis. The presence of publication bias is evaluated by funnel plots. Any statistical test with a *P*-value <0.05 indicate the presence of statistically significant. All of these statistical analyses are performed by R software and Review Manager 5.3.

## Results

### Search Results and Characteristics of Studies

**Figure 1:**
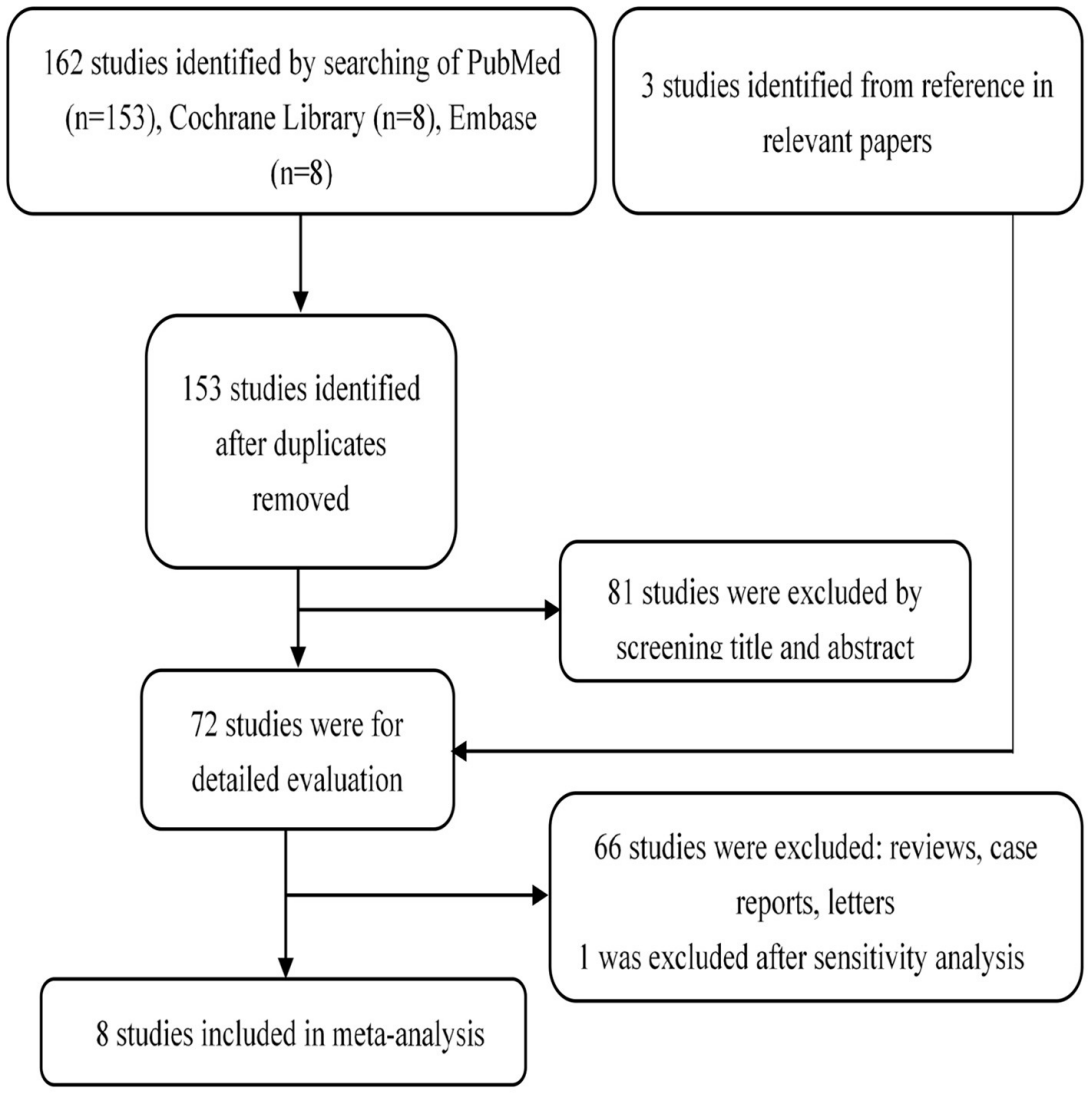
Flow chart demonstrating process of study selection.

One hundred and sixty-nine publications are recruited after being searched on online databases: PubMed (*n* = 153), Cochrane Library (*n* = 8), and Embase (*n* = 8) (Figure 1). A total of 72 full-test studies are assessed as qualified after eliminating duplicates and screening abstracts. We also obtain three additional studies (14, 17, 29) by scanning the reference lists of eligible studies. We exclude one study (30) after sensitivity analysis due to its high heterogeneity. Finally, eight studies (10, 14, 15, 17–19, 29, 31) that all published from 2005 to 2019, containing 230 patients, are included. Two studies (17, 18) were taken in China, two in USA (14, 31), two in UK (10, 29), and the remaining two studies come from Netherlands (15) and Canada (19), respectively (Table 1). The organ involvements in the included patients are show in Supplementary Table 1. Koukoulaki and Jayne (29), Jones et al. (10), Hu et al. (17), Han et al. (18), and Silva et al. (31) referred to patients with resistance to standard therapy or relapse who were treated with MMF as first-line therapy. Joy et al. (14), Stassen et al. (15), and Tuin et al. (19), used this agent as secone-line therapy.

**Table 1:** Basic characteristics of included studies.

**Study**	**Country**	**Study design**	**Cases**	**Disease category (cases, n)**	**ANCA**	**Follow-up months**	**Remission**	**Study quality score**
Joy et al. (14)	USA	Case series	12	WG (7), MPA (2), NCGN (2), CSS(1)	MPO (3) PR3 (9)	12	6/12	5
Koukoulaki and Jayne (29)	UK	Case series	22	AASV	N/A	21.9 ± 14.8^*^	18/22	5
Stassen et al. (15)	Netherlands	Cohort	32	WG (29), MPA (3)	MPO (3) PR3 (29)	12 (2–58) #	CR: 25/32 PR: 6/32	6
Hu et al. (17)	China	Randomized	35	MPA (34), WG (1)	MPO (28) PR3 (2)	6	14/18 (IVC: 8/13)	5
Silva et al. (31)	USA	Cohort	17	MPA	MPO (17)	18	13/17	6
Han et al. (18)	China	Randomized	42	NA	MPO (42)	6	14/18 (IVC: 8/13)	9
Jones et al. (10)	UK	Randomized	140	MPA (49), GPA (91)	MPO (53) PR3 (82)	18	47/70 (IVC: 43/70)	9
Tuin et al. (19)	Canada	Randomized	84	NA	MPO (9) PR3 (75)	48	27/41 (IVC: 35/43)	9

*ANCA, Antineutrophil cytoplasmic antibody; AASV, Antineutrophil cytoplasm antibodies associated systemic vasculitis; IVC, Intravenous cyclophosphamide; WG, Wegener's granulomatosis; MPA, Microscopic polyangiitis; GPA, Granulomatosis with polyangiitis; CSS, Churg–Strauss syndrome; NCGN, Necrotizing and crescentic glomerulonephritis alone; MPO, Myeloperoxidase; PR3, Proteinase 3; CR, Complete remission; PR, Partial remission; NA, Not available*;

# *Median (range)*;

* *Mean ± standard variance*.

Most studies were high-quality (average quality score = 6.75), see Supplementary Table 2 for details. This meta-analysis is carried out according to the guidelines of the Preferred Reporting Items for Systematic Reviews and Meta-Analyses (PRISMA) Statement (Supplementary Table 3) (30).

### Efficacy of MMF in AAV Patients

**Figure 2:**
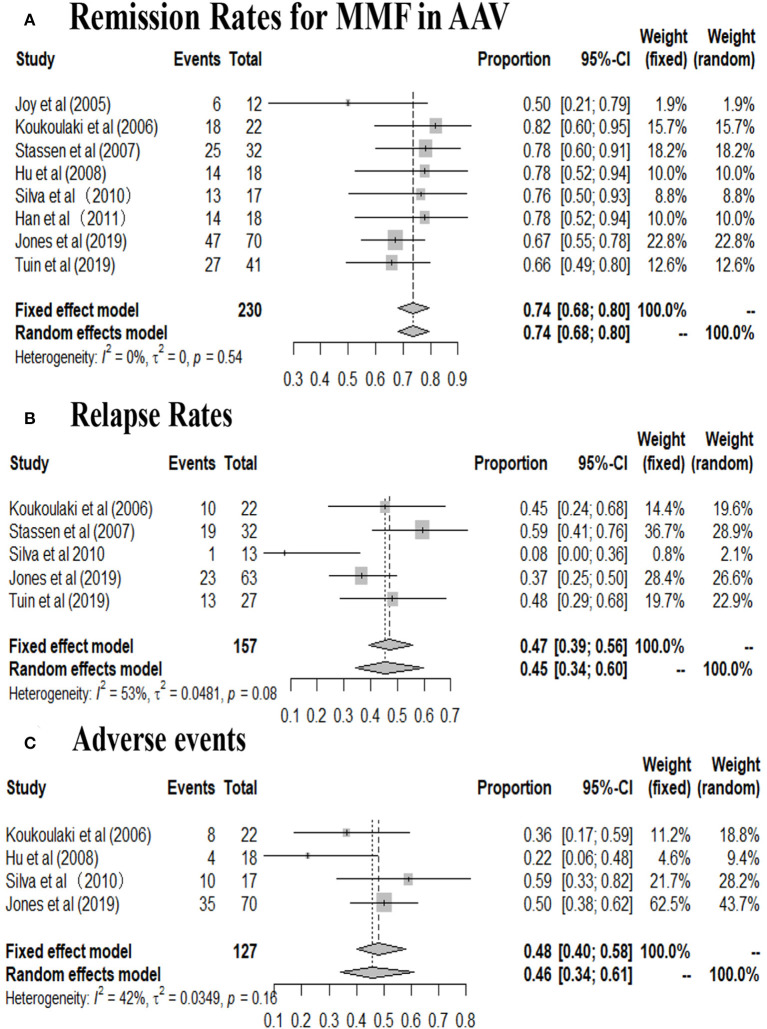
**(A)** Remission rates, **(B)** relapse rates, and **(C)** adverse events in patients undergoing MMF treatment in the included studies (By R software).

**Figure 3:**
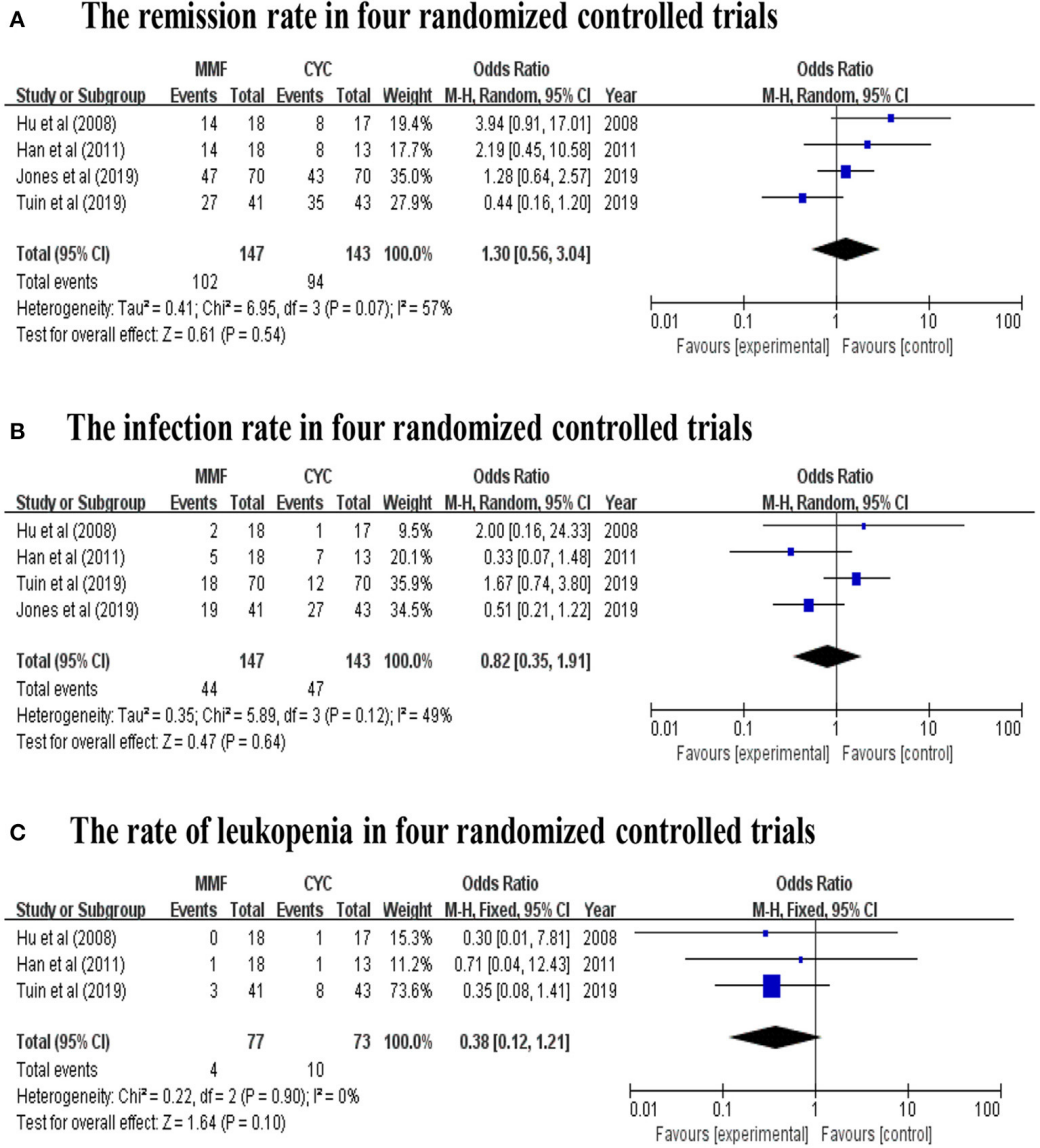
**(A)** The remission rate, **(B)** the infection rate, and **(C)** the rate of leukopenia in four random controlled trials (By Review Manager 5.3).

**Figure 4:**
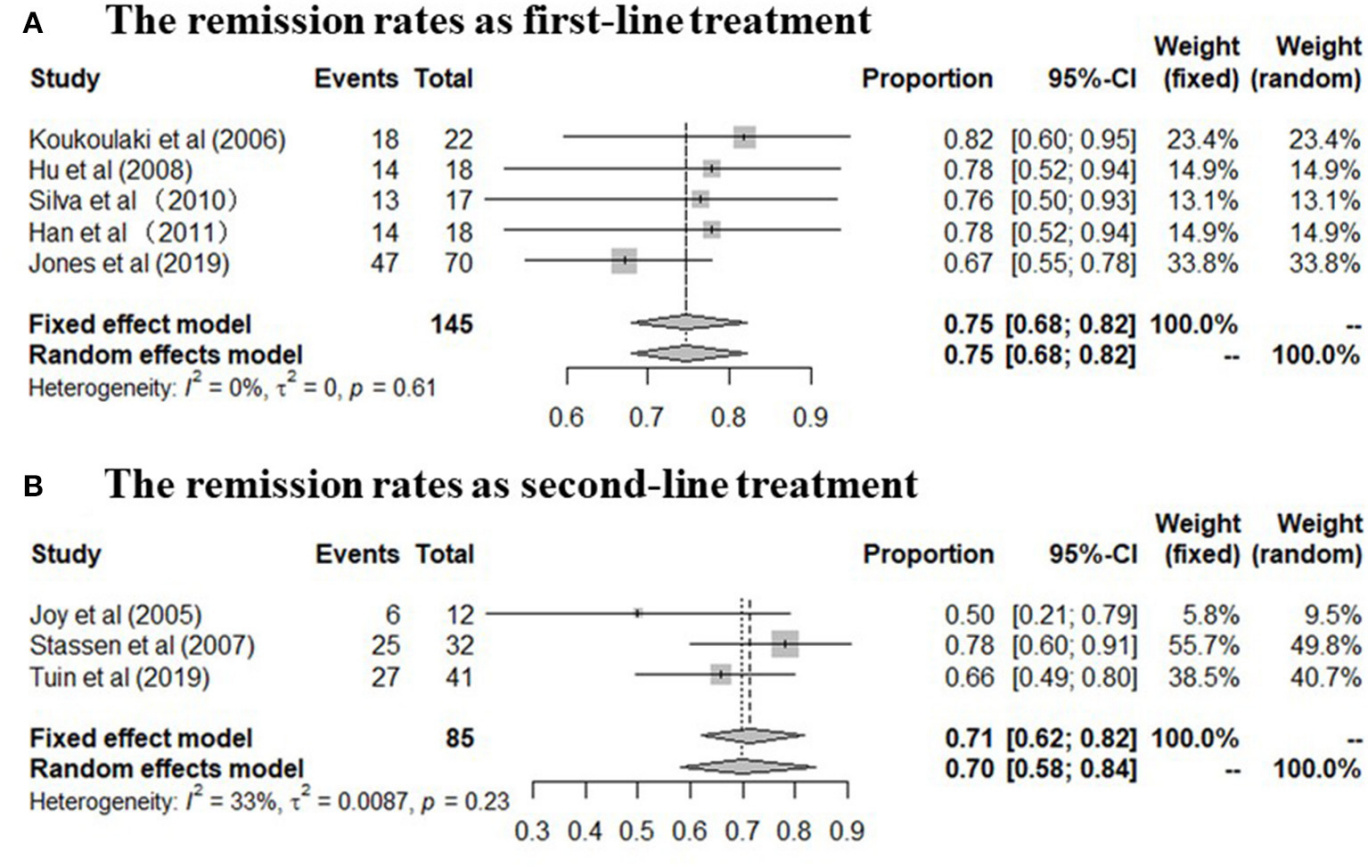
**(A,B)** The remission rate in patients undergoing MMF treatment in the included studies (The forest plots was mapped based on the use of MMF as first-line or second-line treatment).

A total of 230 patients in eight studies (10, 14, 15, 17–19, 29, 31) reported the remission rate of MMF in AAV. Joy et al., Stassen et al., and Tuin et al., referred to patients with resistance to standard therapy or relapse who were treated with MMF as second line therapy. And other five studies used this agent as first line therapy. The remission rates of the included study are between 50 and 82%, and the pooled remission rate is 74.0% (95% CI: 68.0–80.0%) with statistical heterogeneity 0% (tau^2^ = 0, and *P* = 0.54; Figure 2A), according to the fixed effects model. The relapse rates is 45% (95% CI: 34.0–60.0%) with statistical heterogeneity 53% (tau^2^ = 0.0481, and *P* = 0.08; Figure 2B), Four randomized controlled trials (RCTs) (10, 17–19) aimed at comparing the effects of MMF with CYC during induction therapy for AAV include 147 cases in MMF group and 143 cases in CYC group. Heterogeneity test show that the heterogeneity of the remission rate among the studies is significant (*I*^2^ = 57%, *P* = 0.07), and random effect model is used for analysis. Figure 3A show that the remission rate of the two groups have no statistical significance (*Z* = 0.61, *P* > 0.05). Based on the use of MMF as first-line or second-line treatment, we map the forest plots. And the remission rate is shown in Figure 4.

### Efficacy of MMF on Clinical and Laboratory Parameters

Mean dose of MMF per day is shown in Table 2. The effect of MMF on serum creatinine, mean ANCA titres, Birmingham Vasculitis Activity Score (BVAS), estimated glomerular filtration rate, proteinuria, erythrocyte sedimentation rate and C-reactive protein are described in eight studies, respectively, except the study by Koukoulaki and Jayne (29) (Table 2).

**Table 2:** Clinical and laboratory parameters in patients undergoing MMF treatment in the included studies.

**Study**	**At MMF onset**	**At the follow-up period of 6 months**	**Mean does of MMF per day (g/day)**	***P*-vaule**
**Serum creatinine**				
Joy et al. (14)	2.3 ± 2.2 (mg/dL)^*^	1.8 ± 1.2 (mg/dL)^*^	2.0–3.0	0.4679
Stassen et al. (15)	106 (73–542) μmol/l #	CR: 110 (73–500) μmol/l NR and PR: 99 (88–542) μmol/l#	2.0	NA
Hu et al. (17)	3.55 ± 1.1 (mg/dL)^*^	NA	1.5–2.0	NA
Han et al. (18)	318.7 ± 283.4 mol/l^*^	114.9 μmol/l (mean sCr level)	1.0	NA
**Mean ANCA titres**				
Joy et al. (14)	57 ± 29.2 μ/ml^*^	43.3 ± 35.0 μ/ml^*^	2.0–3.0	0.3736
Silva et al. (31)	54 (16–113) EU/ml#	5 (4–10) EU/ml#	1.5–2.0	<0.01
Han et al. (18)	97.6 ± 88.7 μ/ml ^*^	22.4 μ/ml (mean anti-MPO antibody level)	1.0	NA
**BVAS**				
Stassen et al. (15)	14 (5–29)#	CR: 13 (5–29); NR and PR: 15 (6–27)#	2.0	NA
Hu et al. (17)	15.6 ± 3.3^*^	0.2 ± 0.89^*^	1.5–2.0	NA
Han et al. (18)	17.8 ± 4.2^*^	NA	1.0	NA
Jones et al. (10)	19 (13–25)#	NA	2.0–3.0	NA
Tuin et al. (19)	15 (13–19)#	NA	2.0	NA
**CRP**				
Stassen et al. (15)	39 (1–361) mg/l#	CR: 39 (1–311) mg/l; NR and PR: 35 (6–361) mg/l#	2.0	NA
Silva et al. (31)	0.5 (0.3–35.3) mg/dl#	NA	1.5–2.0	NA
Han et al. (18)	35.7 ± 50.2 mmol/l^*^	NA	1.0	NA
Jones et al. (10)	22 (7.5–52) mg/L#	NA	2.0–3.0	NA
Tuin et al. (19)	33 (10–57) mg/L#	NA	2.0	NA
**ESR**				
Silva et al. (31)	38 (24–73) mm/h#	NA	1.5–2.0	NA
Han et al. (18)	76.1 ± 43.0 mm/h^*^	NA	1.0	NA
Jones et al. (10)	54 (31–98) mm/h#	NA	2.0–3.0	NA
**eGFR**				
Silva et al. (31)	46 (34–63) ml/min#	47 (33–72) ml/min#	1.5–2.0	NA
Han et al. (18)	35.5 ± 29.2 ml/min/1.73 m^2^^*^	21.64 ml/min/1.73 m^2^ (mean eGFR level)	1.0	NA
Jones et al. (10)	51 (29–92) ml/min/1.73 m^2^#	NA	2.0–3.0	NA
**Proteinuria**				
Hu et al. (17)	3.05 ± 2.85 (g/24 h)^*^	NA	1.5–2.0	NA
Silva et al. (31)	889 (400–2,208) mg/24 h#	384 (151–1,071) mg/24 h#	1.5–2.0	<0.01
Han et al. (18)	2.21 ± 1.68 (g/24 h) ^*^	1.256 g/24 h (mean proteinuria level)	1.0	NA

# *Median (range)*;

* *Mean ± standard variance; BVAS, Birmingham Vasculitis Activity Score; CRP, C-reactive protein; ESR, erythrocyte sedimentation rate; eGFR, estimated glomerular filtration rate; NA, not available*.

Silva et al. (31) indicated that the patients had statistically lower parameters of mean ANCA titres and proteinuria in the treatment with MMF (*P* < 0.01), comparing with baseline.

### Safety of MMF in AAV Patients

Four studies (10, 17, 29, 31) reported the number of patients with adverse events related to MMF use in AAV patients. The mainly adverse events were infections, gastrointestinal symptoms and leukopenia. Infections, accounting for 35.6%, including pulmonary infection, herpes zoster, and urinary tract infection, chiefly occurred in two studies (10, 29). Gastrointestinal symptoms, accounting for 14.8%, including diarrhea, bloating, abdominal cramping, nausea, vomiting, constipation, and mid-epigastric pain principally occurred in two studies (17, 31). And leukopenia accounts for about 14.8%. Psychological events attributable to MMF were reported in six patients (29). The incidence of adverse events range from 22.0 to 59.0% (95% CI: 34.0–61.0%; Figure 2C). Four studies reported the number of patients with relapse related to MMF use in AAV patients. Four RCTs (10, 17–19) aimed at comparing the efficacy of MMF with CYC during induction therapy in AAV patients show that the infection rate of the two groups has no statistical significance (*Z* = 0.47, *P* > 0.05; Figure 3B). Three (17–19) of the four RCTs reported the number of patients with leukopenia. The forest plot show that the rate of leukopenia of the two groups has no statistical significance (*Z* = 1.64, *P* > 0.05; Figure 3C).

### Publication Bias

**Figure 5:**
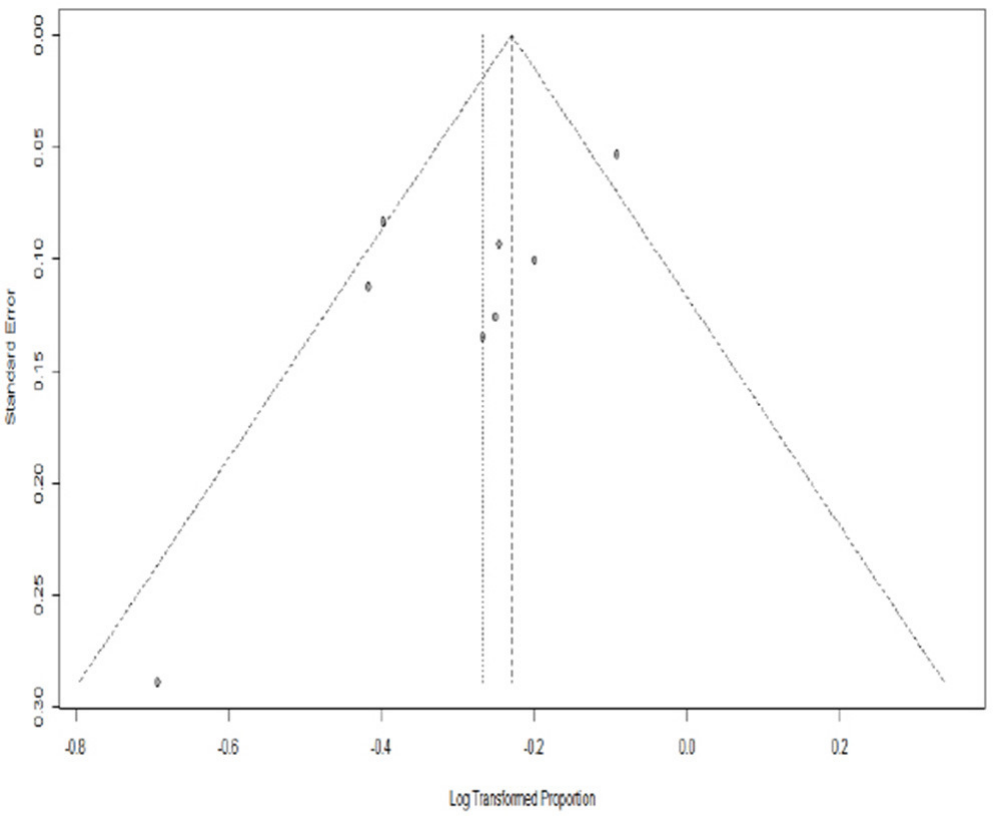
Funnel plot standard error by remission rate for AAV. PLN, log transformation (By R software).

Figure 5 show that there is no significant evidence of publication bias for the remission rates with MMF in AAV patients when the funnel plot is analyzed.

## Discussion

The meta-analysis of the eight studies shows that the remission rate of MMF for induction treatment in patients with AAV is 77.0%. One open-label, multicenter, randomized, controlled trial (4) showed that the remission rate of CYC for induction treatment in patients with AAV was 78.9%, which seemed to be no obvious difference from that of MMF. To improve the level of evidence, we combine the data of four RCTs and find no differences between the efficacies of MMF and CYC for induction treatment in AAV. And the recently published meta analyses (20, 21) included the same four RCTs and their results showed similar efficacy of MMF and CYC with no statistical significance for induction treatment in patients with AAV, which was the same as ours. Moreover, they reached conclusions that there was no difference in the therapeutic effects of MMF and CYC for AAV patients (20), and MMF was equivalent to CYC (21). Does MMF really have the same status as CYC for induction treatment in patients with AAV? Probably not.

Indeed, a full review of current limited trials in MMF is however preferable to an assembly of observations with an erroneous outcome. After more carefully and deeply reviewing the characteristics of the four RCTs and analyzing the existing data, we find that the study by Hu et al. (17) was not able to demonstrate any interventional findings due to its limited patient numbers and high loss in follow up (23% of patients were lost to follow up in the CYC group). One big trial by Tuin et al. (19) was comparing MMF and CYC as re-induction treatment for first or second relapse in patients previously treated with CYC. These patients were not treatment naïve with a potential bias toward CYC non-responder. One (18) of the remaining RCTs was a single-center study from China, and another (10) was a multi-center international trial. It is difficult to mix the remaining single-center study with the multi-center international trial to perform meta-analysis. In addition, error estimation deteriorates with <5 studies in a meta-analysis. Therefore, it is more inappropriate to mix the four RCTs together for analysis owing to the limitations of themselves. Stating that MMF is equal to CYC in inducing remission of AAV based on the results of these four RCTs could be incorrectly interpreted otherwise.

So, what role does MMF play in the induction therapy of AAV? As is known, AAV is a group of diseases with strong heterogeneity (with non-life-threatening and life-threatening patients, different ANCA types and different organs involved, such as lung, kidney, eye, and so on). MMF as an induction therapy in AAV highlights our treatment dilemma that we do not adequately differentiate our patients into risk categories and perform subgrouping. A certain subgroup of patients certainly responds to MMF and it is of great importance to identify these patients to minimize toxicity of CYC. How can we find this subgroup and identify it? There are some phase II trials (18, 31) that showed the potential effects of MMF in AAV, but the phase III trials (10, 19) were negative for induction of remission. This is because these phase II trials and retrospective studies were selecting 100% MPO-ANCA positive AAV patients (18, 31). Moreover, a meta-regression analysis (20) of the four RCTs (10, 17–19) that used MMF as induction therapy showed a positive correlation between the risk ratio of the remission rates and MPO-ANCA. Therefore, MMF may be effective in inducing remission treatment for MPO-ANCA positive AAV subsets. Besides, another characteristic of these phase II trials and retrospective studies is that they included patients with mild to moderate renal involvement (17, 31). In addition, the successful introduction of MMF as a treatment for renal allograft reduced the incidence of acute rejection (7) and it had been used in various immune-mediated nephritis (8, 9). Therefore, we speculate that MMF may be more suitable for patients with immune-mediated renal involvement, such as AAV with mild to moderate renal involvement.

MMF, being the potentially less potent agent in comparison to CYC, may be not suitable for life-threatening AAV patients. CYC and RTX are recommended for remission induction therapy in patients with life-threatening AAV, because RTX has been shown to be as effective as CYC in life-threatening AAV (32). In non-life-threatening AAV, immunotherapy with RTX has however demonstrated similar rates of serious infection, compared to life-threatening AAV (33). That may be because when using RTX in the treatment of immune-related diseases, the occurrence of infection correlated poorly with the types of immune-related diseases and correlated more strongly with the depletion of B-cells, as the use of RTX may cause CD20-expressing B-cells reduction and confer susceptibility to bacterial, viral and fungal infections. Consequently, there is no necessity to use RTX, the strong immunosuppressant, in non-life-threatening AAV patients. Instead, MMF should be used as a complementary drug to CYC, as the immunomodulatory effect of MMF may be less pronounced due to different pharmacokinetics. It is false to conclude that MMF is as effective as CYC in the treatment of AAV as in reality it is only suitable for a subgroup of AAV patients. Patients with MPO-ANCA positive, mild to moderate renal involvement and non-life-threatening state may be included in a subgroup of patients certainly responds to MMF. The false conclusion may be potentially dangerous for the less experience clinicians.

Our study found that the relapse rate of MMF in inducing remission of AAV was as high as 45%, while one big trail by de Groot et al. (4) reported that the rate of CYC was 14.5%. According to the meta-analysis of the four RCTs, MMF, despite being the potentially less potent agent in comparison to CYC, did unfortunately demonstrate similar side effects of infection. Based on the poor performance of MMF in relapse rates and infection rates, it can be confirmed again that we cannot reach the conclusion that MMF has the same status as CYC in the induction treatment of AAV. We sometimes hesitate to use CYC for induction therapy in patients with AAV because of its malignancies and infertility (32). However, it is not the reason why MMF can replace CYC in the induction treatment of AAV. This is because (i) long-term side effects of CYC such as malignancy and infertility were not investigated due to the nature of short follow up of interventional trials; (ii) the peak age of AAV is at 55–64 (34), 65–74 (35), and ≥75 years (36), who may have less need for reproduction.

MMF may be a complementary immunosuppressive agent for induction therapy in mild to moderate renal involvement with MPO-ANCA-positive and non-life-threatening AAV. When assessing the efficacy of new drugs in AAV or comparing the effects of different drugs in AAV, risk stratification (life-threatening or non-life-threatening) and subgrouping (such as different ANCA types and different organs involved) should be carried out first as AAV is a group of diseases with strong heterogeneity.

## Data Availability Statement

The original contributions presented in the study are included in the article/Supplementary Material, further inquiries can be directed to the corresponding author/s.

## Author Contributions

AX conceived this project. AX, CX, YL, and BC designed the study. GY and DL searched the literature data. YS, LH, and YY extracted the data. AX, CX, GY, YS, LZ, ZG, YY, and SS drafted the manuscript. All authors critically reviewed the manuscript and approved the final version of the manuscript. AX, CX, and GY have equally contributed as first authors to this manuscript. GY and YS have equally contributed as senior authors to this manuscript.

## Conflict of Interest

The authors declare that the research was conducted in the absence of any commercial or financial relationships that could be construed as a potential conflict of interest.

## References

[1] Yates M Watts R. ANCA-associated vasculitis. Clin Med (Lond). (2017) 17:60–4. 10.7861/clinmedicine.17-1-60 28148583 PMC6297586

[2] Watts RA Mahr A Mohammad A.J Gatenby P Basu N Flores-Suarez LF. Classification, epidemiology and clinical subgrouping of antineutrophil cytoplasmic antibody (ANCA)-associated vasculitis. Nephrol Dial Transplant. (2015) 30(Suppl 1):i14–22. 10.1093/ndt/gfv022 25805746

[3] Koukoulaki M Iatrou C. The role of mycophenolate in the treatment of antineutrophil cytoplasmic antibody-associated vasculitis. World J Nephrol. (2019) 8:75–82. 10.5527/wjn.v8.i4.75 31523631 PMC6715575

[4] de Groot K Harper L Jayne DR Flores Suarez LF Gregorini G Gross WL. Pulse versus daily oral cyclophosphamide for induction of remission in antineutrophil cytoplasmic antibody-associated vasculitis: a randomized trial. Ann Intern Med. (2009) 150:670–80. 10.7326/0003-4819-150-10-200905190-00004 19451574

[5] Jayne D. Treatment of ANCA-associated systemic small-vessel vasculitis. APMIS Suppl. (2009) 127:3–9. 10.1111/j.1600-0463.2009.02470.x 19515133

[6] Hoffman GS Kerr GS Leavitt RY Hallahan CW Lebovics RS Travis WD. Wegener granulomatosis: an analysis of 158 patients. Ann Intern Med. (1992) 116:488–98. 10.7326/0003-4819-116-6-488 1739240

[7] Halloran P Mathew T Tomlanovich S Groth C Hooftman L Barker C. Mycophenolate mofetil in renal allograft recipients: a pooled efficacy analysis of three randomized, double-blind, clinical studies in prevention of rejection. The International Mycophenolate Mofetil Renal Transplant Study Groups. Transplantation. (1997) 63:39–47. 10.1097/00007890-199701150-00008 9000658

[8] Briggs WA Choi MJ Scheel PJ Jr. Successful mycophenolate mofetil treatment of glomerular disease. Am J Kidney Dis. (1998) 31:213–7. 10.1053/ajkd.1998.v31.pm9469489 9469489

[9] Miller G Zimmerman R 3rd Radhakrishnan J Appel G. Use of mycophenolate mofetil in resistant membranous nephropathy. Am J Kidney Dis. (2000) 36:250–6. 10.1053/ajkd.2000.8968 10922302

[10] Jones RB Hiemstra TF Ballarin J Blockmans DE Brogan P Bruchfeld A. Mycophenolate mofetil versus cyclophosphamide for remission induction in ANCA-associated vasculitis: a randomised, non-inferiority trial. Ann Rheum Dis. (2019) 78:399–405. 10.1136/annrheumdis-2018-214245 30612116

[11] Jebali H Khadhar M Mami I Beji S Sellami M Hassen M. Predictors of renal outcomes in anti-neutrophil cytoplasmic antibody glomerulonephritis. Saudi J Kidney Dis Transpl. (2020) 31:182–90. 10.4103/1319-2442.279939 32129212

[12] Nowack R Gobel U Klooker P Hergesell O Andrassy K van der Woude FJ. Mycophenolate mofetil for maintenance therapy of Wegener's granulomatosis and microscopic polyangiitis: a pilot study in 11 patients with renal involvement. J Am Soc Nephrol. (1999) 10:1965–71. 10477149 10.1681/ASN.V1091965

[13] Langford CA Talar-Williams C Sneller MC. Mycophenolate mofetil for remission maintenance in the treatment of Wegener's granulomatosis. Arthritis Rheum. (2004) 51:278–83. 10.1002/art.20240 15077273

[14] Joy MS Hogan SL Jennette JC Falk RJ Nachman PH. A pilot study using mycophenolate mofetil in relapsing or resistant ANCA small vessel vasculitis. Nephrol Dial Transplant. (2005) 20:2725–32. 10.1093/ndt/gfi117 16188901

[15] Stassen PM Tervaert JW Stegeman CA. Induction of remission in active anti-neutrophil cytoplasmic antibody-associated vasculitis with mycophenolate mofetil in patients who cannot be treated with cyclophosphamide. Ann Rheum Dis. (2007) 66:798–802. 10.1136/ard.2006.060301 17179175 PMC1954648

[16] Souza AWS Calich AL Mariz HA Ochtrop MLG Bacchiega ABS Ferreira GA. Recommendations of the Brazilian Society of Rheumatology for the induction therapy of ANCA-associated vasculitis. Rev Bras Reumatol Engl Ed. (2017) 57 (Suppl 2):484–96. 10.1016/j.rbre.2017.06.003 28754431

[17] Hu W Liu C Xie H Chen H Liu Z Li L. Mycophenolate mofetil versus cyclophosphamide for inducing remission of ANCA vasculitis with moderate renal involvement. Nephrol Dial Transplant. (2008) 23:1307–12. 10.1093/ndt/gfm780 18065810

[18] Han F Liu G Zhang X Li X He Q He X. Effects of mycophenolate mofetil combined with corticosteroids for induction therapy of microscopic polyangiitis. Am J Nephrol. (2011) 33:185–92. 10.1159/000324364 21311184

[19] Tuin J Stassen PM Bogdan DI Broekroelofs J van Paassen P Cohen Tervaert JW. Mycophenolate mofetil versus cyclophosphamide for the induction of remission in nonlife-threatening relapses of antineutrophil cytoplasmic antibody-associated vasculitis: randomized, controlled trial. Clin J Am Soc Nephrol. (2019) 14:1021–8. 10.2215/CJN.11801018 31253599 PMC6625631

[20] Kuzuya K Morita T Kumanogoh A. Efficacy of mycophenolate mofetil as a remission induction therapy in antineutrophil cytoplasmic antibody: associated vasculitis-a meta-analysis. RMD Open. (2020) 6:e001195. 10.1136/rmdopen-2020-001195 32371435 PMC7299518

[21] Song GG Lee YH. Comparative efficacy and safety of mycophenolate mofetil versus cyclophosphamide in patients with active antineutrophil cytoplasmic antibody-associated vasculitis: a meta-analysis of randomized trials. Z Rheumatol. (2020). 10.1007/s00393-020-00803-5. [Epub ahead of print]. 32337635

[22] Cochrane-handbook.org. Oxford: Cochrane Handbook for Systematic Reviews of Interventions Version 5.1.0. (2011). Available online at: http://www.cochranehandbook.org/ (cited February 29, 2020).

[23] Singal AK Fontana RJ. Meta-analysis: oral anti-viral agents in adults with decompensated hepatitis B virus cirrhosis. Aliment Pharmacol Ther. (2012) 35:674–89. 10.1111/j.1365-2036.2011.04990.x 22257108

[24] Singal AK Salameh H Kuo YF Fontana RJ. Meta-analysis: the impact of oral anti-viral agents on the incidence of hepatocellular carcinoma in chronic hepatitis B. Aliment Pharmacol Ther. (2013) 38:98–106. 10.1111/apt.12344 23713520

[25] Freeman MF Tukey JW. Transformations related to the angular and the square root. Ann Math Stats. (1950) 21:607–11. 10.1214/aoms/1177729756

[26] Higgins JP Thompson SG. Quantifying heterogeneity in a meta-analysis. Stat Med. (2002) 21:1539–58. 10.1002/sim.1186 12111919

[27] Higgins JP Thompson SG Deeks JJ Altman DG. Measuring inconsistency in meta-analyses. BMJ. (2003) 327:557–60. 10.1136/bmj.327.7414.557 12958120 PMC192859

[28] Borenstein M Hedges LV Higgins JP Rothstein HR. A basic introduction to fixed-effect and random-effects models for meta-analysis. Res Synth Methods. (2010) 1:97–111. 10.1002/jrsm.12 26061376

[29] Koukoulaki M Jayne DR. Mycophenolate mofetil in anti-neutrophil cytoplasm antibodies-associated systemic vasculitis. Nephron Clin Pract. (2006) 102:c100–7. 10.1159/000089667 16286784

[30] Moher D Liberati A Tetzlaff J Altman DG PRISMA G. Preferred reporting items for systematic reviews and meta-analyses: the PRISMA statement. J Clin Epidemiol. (2009) 62:1006–12. 10.1016/j.jclinepi.2009.06.005 19631508

[31] Silva F Specks U Kalra S Hogan MC Leung N Sethi S. Mycophenolate mofetil for induction and maintenance of remission in microscopic polyangiitis with mild to moderate renal involvement–a prospective, open-label pilot trial. Clin J Am Soc Nephrol. (2010) 5:445–53. 10.2215/CJN.06010809 20093349 PMC2827580

[32] Moher DLA Tetzlaff J Altman DG Grp P. Preferred reporting items for systematic reviews and meta-analyses: The PRISMA statement. Int J Surg. (2010) 8:336–41. 10.1016/j.ijsu.2010.02.007 20171303

[33] Charles P Neel A Tieulie N Hot A Pugnet G Decaux O. Rituximab for induction and maintenance treatment of ANCA-associated vasculitides: a multicentre retrospective study on 80 patients. Rheumatology (Oxford). (2014) 53:532–9. 10.1093/rheumatology/ket381 24282319

[34] Gonzalez-Gay MA Garcia-Porrua C Guerrero J Rodriguez-Ledo P Llorca J. The epidemiology of the primary systemic vasculitides in northwest Spain: Implications of the Chapel Hill Consensus Conference definitions. Arthritis Rheum. (2003) 49:388–93. 10.1002/art.11115 12794795

[35] Watts RA Lane SE Bentham G Scott DGI. Epidemiology of systemic vasculitis: A ten-year study in the United Kingdom. Arthritis Rheum. (2000) 43:414–9. 10.1002/1529-0131(200002)43:2<414::AID-ANR23>3.0.CO;2-0 10693883

[36] Mohammad AJ Jacobsson LT Westman KW Sturfelt G Segelmark M. Incidence and survival rates in Wegener's granulomatosis, microscopic polyangiitis, Churg-Strauss syndrome and polyarteritis nodosa. Rheumatology (Oxford). (2009) 48:1560–5. 10.1093/rheumatology/kep304 19797309

